# Synergistic Antitumor Effects of Ivermectin and Metformin in Canine Breast Cancer via PI3K/AKT/mTOR Pathway Inhibition

**DOI:** 10.3390/cimb47060403

**Published:** 2025-05-29

**Authors:** Huili Feng, Lixin He, Talha Umar, Xiao Wang, Wenxuan Li, Bohan Zhang, Xinying Zhu, Ganzhen Deng, Changwei Qiu

**Affiliations:** Department of Clinical Veterinary Medicine, College of Veterinary Medicine, Huazhong Agricultural University, Wuhan 430070, China; 2021302039008@webmail.hzau.edu.cn (H.F.); talhaumar1532@hotmail.com (T.U.); ganzhendeng@sohu.com (G.D.)

**Keywords:** metformin, ivermectin, ROS, PI3K/AKT/mTOR, autophagy

## Abstract

Ivermectin (IVM) is a macrolide antiparasitic drug, and Metformin (MET) is a biguanide oral hypoglycemic drug. Studies have shown that both of them have obvious anti-tumor effects, but there have been no reports on the combined treatment of Canine breast tumors. This report aimed to investigate the effectiveness and the possible mechanism of drug combination on Canine breast cancers. Mouse breast tumor cells (4T1) and canine breast tumor cells (CMT-1211) were, respectively, treated with IVM, MET, and their combination, and then cell viability was assessed. After that, transcriptomic analysis was performed to study the action pathway of the drug combination with regard to its anti-tumor effects. Reactive oxygen species (ROS) levels were detected by flow cytometry, and autophagosome formation was observed by transmission electron microscopy (TEM). Immunofluorescence detected the cytoplasmic translocation of LC3B and P62 into the nucleus. Western blot detected the protein expressions of LC3B, P62, Beclin1, Bcl-2, p-PI3K, p-AKT, and p-mTOR. Our transcriptomic analysis showed that the combination of IVM and MET regulated the expression of autophagy-related genes and pathways, including the PI3K/AKT/mTOR signaling pathway. Our in vitro experiments showed that the combination of two drugs had a considerably significant effect on cytotoxicity, ROS levels, and the formation of autophagosomes compared to each drug alone. Meanwhile, the in vivo experiments showed that IVM combined with MET had an obvious inhibitory effect on tumor growth in canine breast tumor xenografts. This study concluded that IVM with MET activated autophagy, which killed breast cancer cells by inhibiting the activation of the PI3K/AKT/mTOR pathway and promoting the excessive accumulation of ROS. It offers a theoretical foundation for the synergistic effects of MET and IVM to suppress breast cancer cell activity.

## 1. Introduction

Breast cancer is one of the most common malignant tumors in humans and animals. Breast cancer has a high rate of morbidity and mortality [1,2]. Drug resistance in advanced breast cancer limits the therapeutic effectiveness of treatment [3]. Drug combination application is one of the crucial strategies to develop new cancer therapies and solve multi-drug resistance [4], and the “new use of old drugs” can reduce the development risk cost and shorten the development time [5]. IVM is a broad-spectrum antiparasitic drug that has recently been shown to exhibit strong anticancer action. This effect may be mediated by a variety of pathways that impact the proliferation and metastasis of cancer cells. IVM can treat various kinds of cancer, including breast cancer [6], ovarian cancer [7], colon cancer [8], carcinoma of the urinary bladder [9], and so on, Diao H et al. found that ivermectin inhibited the growth of canine breast tumor cell lines in a dose-and time-dependent manner, and the significant inhibition of ivermectin on tumor growth was observed in canine breast tumor xenografts [10].

In the treatment of type 2 diabetes, MET is a first-line medication. It plays a hypoglycemic role by inhibiting gluconeogenesis and glucose synthesis in the liver by activating AMPK signal transduction in liver cells [11]. Its hypoglycemic mechanism is similar to that of the anti-tumor mechanism, and both type 2 diabetes and breast cancer are metabolic diseases. It has been in clinical use for decades, and its safety has been widely recognized. Recent studies have shown that MET exerts anti-tumor effects in various types of cancer, such as breast cancer [12], thyroid cancer, [13]^,^ and prostate cancer [14]. Fan Y. et al. found that metformin could inhibit the proliferation of canine breast tumor (CMGT) cells through cell cycle arrest, the induction of apoptosis, the activation of AMPK, and the inhibition of AKT/mTOR signaling pathway [15]. However, the body may resist the effects of anti-tumor medications in tumor patients treated with a single medication for an extended period due to gene mutations, changes in protein expression, and loss of stem cell target expression, which presents serious hurdles for the treatment of cancer [16].

On the contrary, the new target or protein expression regulation changes initiated by the drug combination may avoid the body’s tolerance to the drug, improve efficacy, and reduce toxicity [17]. From the perspective of cell homeostasis, autophagy is one of the important mechanisms for coping with oxidative stress. Autophagy can reduce cell damage by clearing ROS, damaged biomoleculins, and mitochondria to meet cells’ metabolic needs and cope with specific stress states [18]. However, when ROS accumulation is excessive, or the duration is too long, autophagy may not be able to clear excess ROS, resulting in a large number of mitochondrial autophagy and insufficient cell energy, which will lead to autophagic cell death [19].

This study investigates, for the first time, the combined use of IVM and MET to determine whether the excessive accumulation of intracellular ROS inhibits the activation of the PI3K/AKT/mTOR signaling pathway, inducing a significant number of autophagosomes in breast cancer cells. This process ultimately leads to autophagic cell death, providing a new basis for the potential use of IVM combined with MET as an autophagy inducer and a novel treatment method for breast cancer.

## 2. Materials and Methods

### 2.1. Reagents

IVM (purity ≥98%) was purchased from Sigma Company, and MET (purity ≥98%) was purchased from Shanghai Yuanye Biotechnology Co., Ltd. The use of primary antibodies as follows: β-actin (ABclonal, AS014), LC3B (ABclonal, A19665), Beclin1 (ABclonal, A21191), P62 (protein tech, 18420-1-AP), Bcl-2 (ABclonal, A19693), PI3 Kinase p85 (ABclonal, A4992), Phospho-PI3K P85α (ABclonal, Abclonal, AP0854), AKT (ABclonal, A20779), Anti-AKT (Wanleibio, WLP001), mTOR (ABclonal, A11345), and Phospho-mTOR (ABclonal, AP0115). HRP goat anti-rabbit protein (no clone), Alexa Fluor 594-goat anti-rabbit protein (Abbox, Jiangsu, China), Cy3 goat anti-rabbit protein (H + L) (no clone), and FITC goat anti-rabbit protein (Wuhan, China) were used as secondary antibodies for Western blot or immunofluorescence.

### 2.2. Cell Culture

The CMT-1211 cell line used in this study was isolated from a canine breast tumor and donated by Professor Lin Degui, China Agricultural University laboratory. The 4T1 cell line was obtained from the Cell Bank of the Chinese Academy of Sciences (Shanghai, China). All cell lines were grown in DMEM medium (Gibco) containing 10% fetal bovine serum (VISTECH) and 2% penicillin–streptomycin (Sigma) and cultured in a cell incubator at 37 °C and 5%CO_2_ saturated humidity. When the cell density reached more than 80%, the cells were either passaged or used for the next experiment.

### 2.3. Cell Viability Was Detected by the CCK-8 Method

Suspended CMT-1211 and 4T1 cells were inoculated in 96-well plates with 100 μL per well, ensuring at least 2 × 10^3^ cells per well. Five replicates were set for each drug concentration. Cells were cultured in a 5% CO_2_ incubator at 37 °C for 12 h until they reached 50% confluence. Different concentrations of IVM, MET, and IVM + MET were then added. After 12, 24, or 48 h of further incubation, the culture medium was replaced with DMEM and CCK-8 solution (9:1 ratio, 100 μL per well). Plates were incubated at 37 °C for 30 min, and absorbance was measured using a Bio-Rad enzyme-labeled instrument with optical density at 450 nm.

### 2.4. Scratch Test

Horizontal lines were marked on the bottom of a six-well plate. A single-cell suspension was inoculated into each well (2 mL per well) and cultured in a CO_2_ incubator at 37 °C for 24 h. After 24 h cells formed a monolayer, vertical lines were drawn with a 200 μL yellow pipette tip, and cells were washed three times with PBS to remove detached cells. Serum-free DMEM, IVM, MET, and IVM+MET solutions were added, and cells were cultured for another 24 h. Cell proliferation was observed using an inverted microscope (Olympus, CKX41, Tokyo, Japan), and the scratch area was analyzed using ImageJ software (https://imagej.net/ij/).

### 2.5. Cell Invasion Experiment

Prepare a 24-well plate and Transwell chamber. Dilute glue A (1×) 12 times with buffer C (0.5×) at 37 °C (Biozellen Inc., Los Angeles, CA, USA). Add 100 µL of this diluted glue A to each upper chamber and incubate at 4 °C for 2 h. Suspend 7.5 × 10^4^ cells, treated with 0, IVM, MET, or IVM+MET for 24 h in 100 µL culture medium. Add 1 mL DMEM with 10% fetal bovine serum to the upper and lower chambers to promote cell migration and invasion. After incubating the plates in a 5% CO_2_ incubator at 37 °C for 24 h, remove the medium, wash twice with PBS, fix with 1 mL 100% methanol for 30 min, and stain with 1 mL 0.1% crystal violet for 30 min. Wash twice with PBS, place the upper chamber upside down on a slide, and observe cell migration under an inverted light microscope.

### 2.6. RNA Sequencing Analysis

To investigate the response mechanism of CMT-1211 and 4T1 cells to IVM, MET, and IVM+MET, Wuhan Kangce Test Technology Co., Ltd. (Wuhan, China) conducted 12 eukaryotic UID-mRNA sequencing analyses (RNA-Seq). The library was constructed and sequenced using an Illumina kit. Data quality control was performed using fastp (version 0.23.0).

Genes with an absolute logFC > 1 and q < 0.05 were considered differentially expressed. Gene enrichment analyses were conducted using GO (Gene Ontology) and KEGG (Kyoto Encyclopedia of Genes and Genomes).

### 2.7. Western Blot for Autophagy-Related Proteins

IVM, MET, and IVM+MET were introduced to CMT-1211 and 4T1 cell lines after they were administered with 2 × 10^5^ cells per well in 6-well plates and grown for an additional night. After a day, the cells were centrifuged for 20 min at 12,000 rpm after being lysed on ice for 30 min in RIPA buffer containing 1% PMSF. A BCA protein quantification kit was used to measure the protein concentration. Samples were electrophorized using SDS-PAGE, then transferred to a PVDF membrane and blocked for 2 h with 5% skim milk. After an overnight incubation period at 4 °C, primary antibodies were 3× washed with TBST. For 2 h, the secondary antibody was incubated at room temperature. The fusion gel imaging equipment was used to take pictures of protein expression, and ImageJ was used to analyze the results.

### 2.8. Transmission Electron Microscope (TEM)

Cells (CMT-1211 and 4T1) were immobilized in 4% glutaraldehyde (Sigma). Prepare ultra-thin slices after dewatering with the sorvallMT5000 (Dupont Instruments, MT5000). The autophagy vacuoles in the cytoplasmic region were calculated using ImageProPlus v.3, stained with lead citrate or 1% uranyl acetate. After IVM, MET, and IVM+MET treated cells for 24 h, (2 × 10^5^)~(1 × 10^6^) cells were collected, and the supernatant was discarded by centrifugation. Wash with PBS once, centrifuge, and discard the supernatant. Fix 2.5% glutaraldehyde above 20 times the sample volume with PBS for 4 h, rinse with 0.1 moL/PBS (dosage 0.5–1.0 mL) twice for 15 min each time, fixed with 1% osmic acid fixing solution for 1 h, and rinse with ddH_2_O twice for 10–15 min each time. The cells were fixed/stained with 2% uranium acetate for 30 min, then dehydrated by ethanol gradient, permeated, embedded, and polymerized. The cells were sliced with an ultrathin micrograph and stained with uranium acetate and lead citrate. Autophagosome formation was observed under TEM.

### 2.9. Measurement of Intracellular ROS Production

ROS production in CMT-1211 and 4T1 cells was measured using a ROS assay kit (Hyhcezmbio, Wuhan, China). Cells were inoculated in 6-well plates at 1 × 10^5^ cells/well and cultured for 24 h. After treatment, cells were collected, centrifuged at 1200 rpm for 5 min, washed twice with PBS, and resuspended in 1 mL of 50 mg/mL DCFH-DA probe per well. Cells were incubated at 37 °C in a CO_2_ incubator for 35 min, then centrifuged, washed with PBS, and resuspended. Rosup (50 mg/mL) was used as a positive control. At least 10,000 live cells were analyzed using flow cytometry (CytoFLEX, Huazhong Agricultural University), and the results were analyzed using FlowJo v10 software.

### 2.10. In Situ Breast Cancer Model in Mice

Balb/c mice female (5–6 weeks) were purchased from the Experimental Animal Center of Huazhong Agricultural University and acclimated for one week. The Animal Ethics Committee of Huazhong Agricultural University approved all studies (HZAUMO-2024-0222). CMT-1211 cells suspended in PBS were transplanted into the fourth mammary fat pad of Balb/c mice to establish the in-situ breast cancer model. Once tumor volume reached 100 mm^3^, mice were randomly divided into 6 groups (5 mice each) and treated with PBS (0.1 mL), IVM (0.2 mg/kg, Sigma, Wuhan, China), MET (100 mg/kg, Shanghai Maclin), IVM+MET (0.1 mg/mL IVM + 50 mg/kg MET), or paclitaxel (10 mg/kg). IVM was administered subcutaneously (0.1 mL/mouse every 2 days), and MET was given via intragastric administration (0.1 mL/mouse daily). Tumor volume (V = 0.5 × length × width^2^) and weight were measured every 2 days. After four weeks, mice were euthanized, and tumor tissue and internal organs were either frozen in liquid nitrogen or fixed in 4% paraformaldehyde for further analysis.

### 2.11. H&E Dyeing

The tissue organs (heart, liver, spleen, lung, and kidney) and mouse mammary tumors were dehydrated, fixed in formaldehyde for 48 h, and then embedded in paraffin wax. After that, they were sliced into 5 μm slices for staining with hematoxylin and eosin (H&E). To capture images, an optical microscope is employed.

### 2.12. Immunofluorescence

CMT-1211 and 4T1 cells were cultured and treated with IVM, MET, and IVM+MET. Following a 24 h incubation period at 37 °C in a 5% CO_2_ incubator, the cells were rinsed with PBS, treated with 4% paraformaldehyde for 30 min to fix them, permeabilized with 0.2% Triton X-100 for 15 min, and then blocked with 5% BSA for 2 h. The cells were incubated with primary antibodies (LC3B and P62), followed by incubation with FITC-labeled or Cy3-labeled goat anti-rabbit IgG for 2 h in a dark environment. The cells were subjected to DAPI staining for a duration of 8 min and were thereafter rinsed with PBS after each stage. Alternatively, tumor sections were treated with xylene and a mixture of ethanol with varying concentrations to remove the wax. Then, they were submerged in a solution containing citric acid to restore the antigen, with a pH level of 6.0. Finally, the sections were heated in a microwave to retrieve the antigen. Sections were subjected to identical methods such as cellular immunofluorescence. The images were observed using a fluorescence microscope (Shanghai, China).

### 2.13. Statistical Analysis

The statistical analysis was conducted using GraphPadPrism8. The data are presented as the mean ± standard deviation (Mean ± SD) of a minimum of three independently conducted studies. Group differences were determined using either bidirectional variance analysis or unpaired *T*-test.* *p* < 0.05,* *p* < 0.01, *** *p* < 0.001,**** *p* < 0.0001 indicates statistical significance.

## 3. Results

### 3.1. Effect of IVM and MET on Breast Cancer Cell Growth

**Figure 1:**
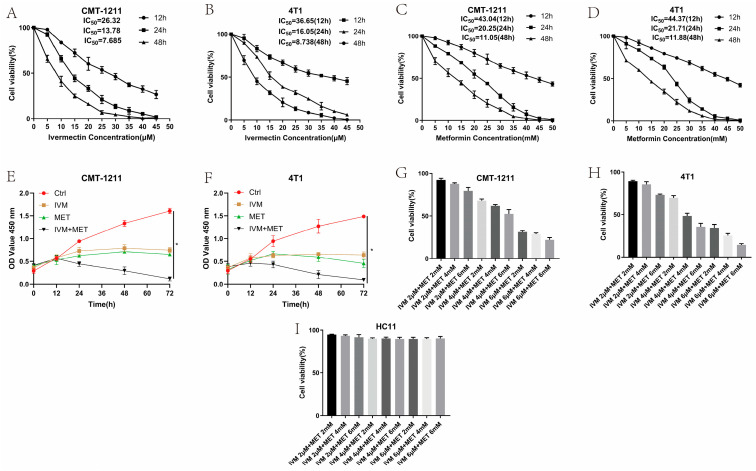
Cytotoxic effects of IVM, MET, and their combination on CMT-1211 and 4T1 cells. (**A**,**B**) CCK-8 assay detected CMT-1211 and 4T1 cell viability at 12 h, 24 h, and 48 h after IVM treatment. (**C**,**D**) CCK-8 assay detected CMT-1211 and 4T1 cell viability at 12 h, 24 h, and 48 h after MET treatment. (**E**,**F**) CCK-8 was used to evaluate the activity of CMT-1211 and 4T1 cells after 0 h, 12 h, 24 h, 36 h, 48 h, 60 h, and 72 h in different treatment groups. (**G**,**H**) IVM of 2 μM, 4 μM, and 6 μM were combined with MET of 2 mM, 4 mM, and 6 mM, respectively. CCK-8 method was used to evaluate the cell viability of CMT-1211 and 4T1 after treatment with different drug groups. (**I**) Cell viability of HC11 control cells.

To assess the impact of IVM, MET, and their combination on breast cancer cell growth, we used the CCK-8 assay with two breast cancer cell lines (CMT-1211 and 4T1). As shown in Figure 1A–D, treatment with IVM or MET significantly reduced the cell viability in both cell lines within 24 h, and the effects depended on the time and dosages. Additionally, the combined treatment of IVM and MET demonstrated a strong inhibited synergistic effect on cell growth than either drug alone (Figure 1E,F). Based on these findings, the optimal concentrations for further studies were determined to be 6 μM IVM and 6 mM MET (Figure 1G,H). To determine whether the combination of the two drugs has a cytotoxic effect on normal breast tumors, we selected normal mouse breast cells HC11 and used the same concentration of drugs. The results demonstrate that not all treatment dosages significantly affect the viability of these non-tumorigenic cells among all the drug treatment groups, confirming that the observed effects were specific to the transformed cells (Figure 1I). Taken together, these results suggested the most excellent inhibited efficacy of IVM combined with MET on breast cancer cells in vitro.

### 3.2. Inhibition of Breast Cancer Cell Migration and Invasion by IVM and MET

**Figure 2:**
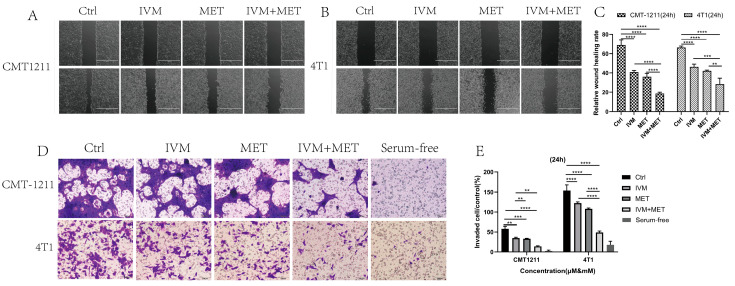
The effects of IVM combined with MET on the migration and invasion of breast cancer cells. (**A**–**C**) Wound healing test was used to measure the migration capacity of tumor cells for 24 h on A scale of 1000 µm. (**D**,**E**) The invasive ability of CMT-1211 and 4T1 cells treated with different treatment groups for 24 h was determined using Transwell chambers precoated with matrix gel. Scale: 100 µm. ** *p* < 0.001, *** *p* < 0.0001, **** *p* < 0.00001.

In drug development, it is crucial to design drugs that prevent cancer cell metastasis and assess their effects on cell migration and invasion [20]. This study examined how IVM and MET combined treatment affects these processes. The cell scratch assay showed that the IVM+MET combination significantly inhibited cell migration compared to either drug alone (Figure 2A–C). In Transwell assays, IVM+MET notably reduced the number of tumor cells, indicating a potent inhibition of cell invasion (Figure 2D–E). These findings demonstrate that combining IVM and MET effectively reduces the migration and invasion of breast cancer cells in vitro.

### 3.3. Transcriptomic Analysis Showed That IVM and MET Regulated Autophagy-Related Pathways

**Figure 3:**
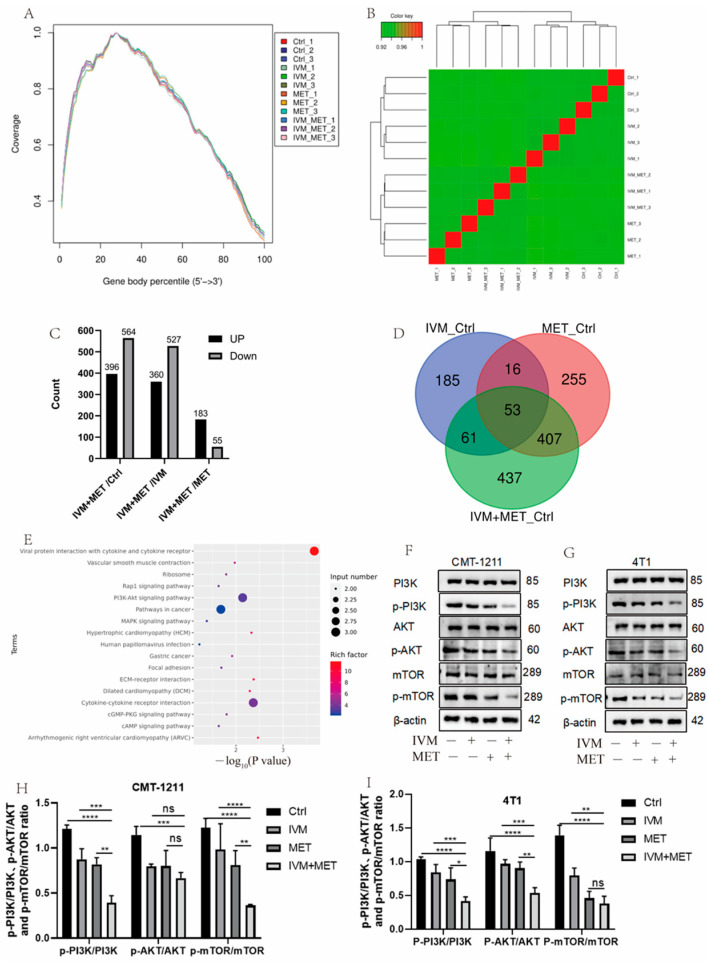
Analysis of transcriptome results. (**A**) The uniform distribution curve of the reads on the reference genome. (**B**) Hierarchical clustering diagram of gene expression levels in samples. (**C**) Differentially expressed genes were screened by IVM+MET/Ctrl group, IVM+MET/IVM group, IVM+MET/MET group. (**D**) Venn diagram of differentially expressed genes. (**E**) KEGG enrichment pathway. (**F**–**I**) Western blotting was used to analyze the expression levels of different groups of PI3K, p-PI3K, AKT, p-AKT, mTOR, and p-mTOR in CMT-1211 and 4T1 cells. All the experiment results are the mean ± standard deviation of three independent experiments. * *p* < 0.05; ** *p* < 0.001; *** *p* < 0.0001; **** *p* < 0.00001; ns = not significant.

To determine the mechanism of IVM combined with MET, transcriptomic analysis was performed, and sequencing uniformity showed that reads were well homogeneous across the reference genome (Figure 3A). Sample biological repeat correlation tests showed that all samples were separated and grouped together (Figure 3B). A total of 960 differential genes were screened in IVM+MET and Ctrl groups, including 396 up-regulated genes and 564 down-regulated genes; 887 differential genes were screened in IVM+MET and IVM groups, including 360 up-regulated genes and 527 down-regulated genes. A total of 238 differential genes were screened in IVM+MET and MET groups, including 183 up-regulated genes and 55 down-regulated genes (Figure 3C). The Venn diagram shows 53 common differential genes (Figure 3D). KEGG analysis showed that IVM combined with MET mainly involved in the PI3K/AKT signaling pathway (Figure 3E). The PI3K/AKT/mTOR pathway is closely related to cell proliferation, differentiation, metabolism, apoptosis, autophagy, and other functions. Therefore, we explored the phosphorylation level of the PI3K/AKT/mTOR signaling pathway. Western blot results showed that, compared with the single medication group, the expressions of p-PI3K, p-AKT, and p-mTOR in the two cells showed a significant downregulation (Figure 3F–H) after combination therapy, indicating that IVM+MET could significantly inhibit the activation of PI3K/AKT/mTOR signaling pathway. It is concluded that the results were consistent with the transcriptional analysis.

### 3.4. IVM Combined with MET-Induced Autophagy of Breast Cancer Cells

**Figure 4:**
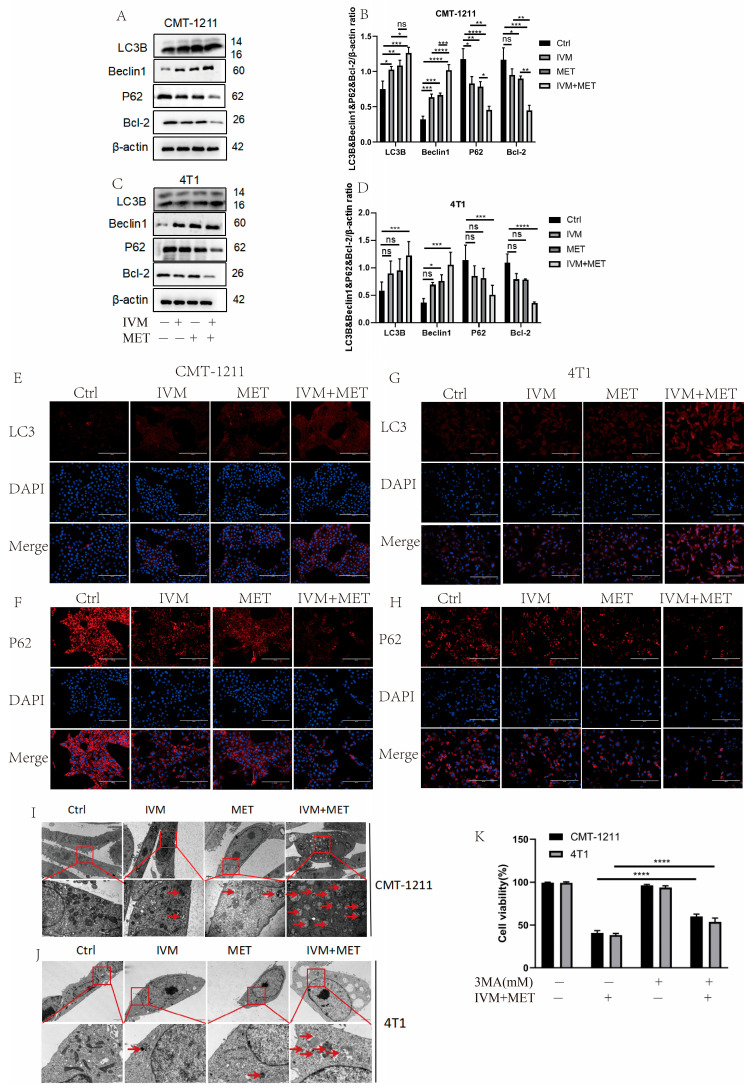
IVM, in combination with MET, promotes autophagy in breast cancer cells. (**A**–**D**) After the cells were treated with different drug combinations for 24 h, the expression levels of LC3B, Beclin1, P62, and Bcl-2 were analyzed by Western blot. (**E**–**H**) Immunofluorescence staining detected the LC3B and P62 expression levels of CMT-1211 and 4T1 cells after 24 h treatment with different drug methods. Scale: 200 µM. (**I**,**J**) TEM was used to observe the autophagosomes of CMT-1211 and 4T1 treated with different drug groups. Red boxes and red arrows pointed to the autophagosomes. 6000× Bar: 2 µM; 60,000× Bar: 200 nm. (**K**) After pre-treating the cells with 3-methyladenine (3-MA) and the combination group, the cell viability of CMT-1211 and 4T1 was detected by the CCK-8 kit. * *p* < 0.05; ** *p* < 0.001; *** *p* < 0.0001; **** *p* < 0.00001; ns = not significant.

Most of the studies have shown that drug-induced autophagy is essential in anticancer therapy [21]. Aberrant autophagy is also an important mechanism of drug resistance in tumors. Therefore, based on transcriptomic analysis, this study examined the regulation of IVM combined with MET on autophagy. Western blot results showed that the protein expression levels of LC3B and Beclin1 in the IVM+MET group were significantly increased. In contrast, the expression levels of P62 and Bcl-2 were significantly decreased (Figure 4A–D). The immunofluorescence results of LC3B and P62 were consistent with the Western blot results (Figure 4E–H). These results indicated that the combination of the two drugs was involved in regulating the autophagy process of breast cancer cells and inducing the occurrence of excessive autophagy. In addition, we observed by TEM that the autophagosome/autophagolysosome accumulation was more significant in the IVM combined MET group than in the control cells of the four treatment groups (Figure 4I–J). At the same time, we pretreated CMT-1211 and 4T1 cells with autophagy blocker 3-MA for 2 h and then pretreated cells with the combined treatment group for 24 h, detected the cell viability, and found that the cell viability of the 3-MA pretreated group was significantly increased (Figure 4K). These data suggest that IVM combined with MET stimulates autophagy in breast tumor cells.

### 3.5. IVM Combined with MET Induces Autophagy of Breast Cancer Cells by Promoting the Overproduction of ROS

**Figure 5:**
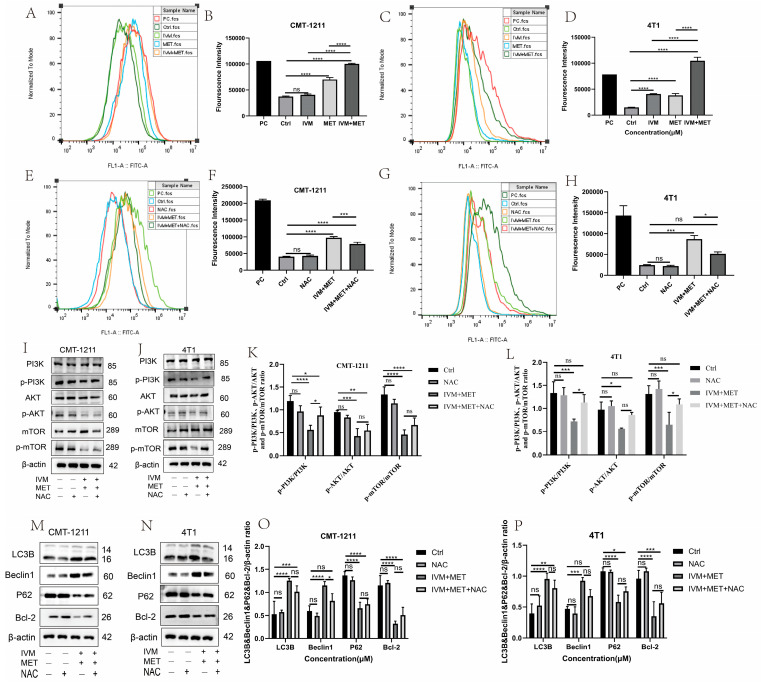
IVM + MET induces autophagy in CMT-1211 and 4T1 by promoting excessive ROS accumulation. (**A**,**B**) Flow cytometry was used to detect the ROS levels of CMT-1211 cells after different treatment groups. PC (Red),Ctrl (Dark Green),IVM (Light Green),MET (Light blue),IVM + MET (Orange).(**C**,**D**) Flow cytometry was used to detect the ROS levels of 4T1 cells after different treatment groups. PC (Red),Ctrl (Light Green), IVM (Orange), MET (Light Blue), IVM + MET (Dark Green). (**E**,**F**) After being treated with 0 mM, NAC (10 mM), IVM + MET, and IVM + MET + NAC, the ROS levels of CMT-1211 cells were detected by flow cytometry. PC (Light Green), Ctrl (Light Blue), NAC (Red), IVM + MET (Orang),IVM + MET + NAC (Dark Green). (**G**,**H**) After being treated with 0 mM, NAC (10 mM), IVM + MET, and IVM + MET + NAC, the ROS levels of 4T1 cells were detected by flow cytometry. PC (Dark green), Ctrl (Light Blue), NAC (Orange), IVM + MET (Light Green), IVM + MET + NAC (Red). (**I**–**L**) After 0 µM, NAC (10 mM), IVM+MET, IVM+MET+NAC, Western blotting was used to analyze the expression levels of PI3K, pPI3K, AKT, p-AKT, mTOR, and p-mTOR in CMT-1211 and 4T1 cells. (**M**–**P**) After 0 µM, NAC (10 mM), IVM+MET, IVM+MET+NAC, Western blotting was used to analyze the expression levels of autophagy-related proteins LC3B, Beclin, P62, and Bcl-2 in CMT-1211 and 4T1 cells. * *p* < 0.05; ** *p* < 0.001; *** *p* < 0.0001; **** *p* < 0.00001; ns = not significant.

The relationship between ROS and autophagy is complex. To prove whether IVM combined with MET influenced the intracellular ROS production, we measured the ROS levels in each experimental group by flow cytometry. The results showed that, compared with the control group, the ROS level in the IVM combined with the MET group was significantly increased (*p* < 0.0001) (Figure 5A–D), and that the free radical scavenger N-acetyl-L-cysteine (NAC) could significantly alleviate the excessive accumulation of ROS caused by IVM combined with MET. The results showed that NAC could partially eliminate IVM combined with MET-induced cytotoxicity (Figure 5E–H). Studies have shown that ROS can regulate the activation level of the PI3K/AKT/mTOR signaling pathway. In this study, the expression of essential proteins in this pathway was detected by Western blot. The results showed that p-PI3K, p-AKT, and p-mTOR were partially restored in the IVM+MET+NAC group compared to the IVM+MET group (Figure 5I–L). To determine whether ROS has a role in IVM combined with MET-induced autophagy, we detected the expression levels of autophagy-related proteins LC3B, Beclin1, P62, and Bcl-2. The results showed that NAC could partially reduce the expression of LC3B and Beclin1 compared with the combination treatment group. The expression of P62 and Bcl-2 was partially restored (Figure 5M–P), suggesting that ROS regulates IVM combined with MET-induced autophagy. These results indicate that IVM+MET can promote ROS overproduction to inhibit PI3K/AK/mTOR activation, thereby leading to autophagy in breast cancer cells.

### 3.6. IVM Combined with MET Can Inhibit the Growth of Breast Cancer In Vivo

**Figure 6:**
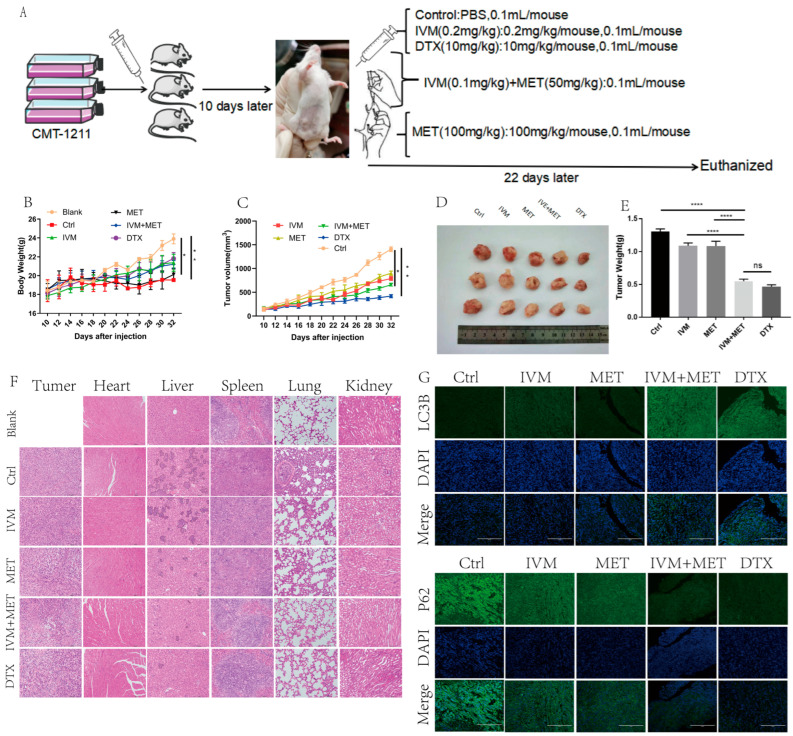
IVM combined with MET can inhibit the growth of canine breast tumor xenografts in vivo. (**A**) Establishing a mouse tumor model and a schematic diagram of the treatment plan. (**B**) Changes in the body weight of mice in each group during treatment. (**C**) Changes in tumor volume of mice in each group during treatment. (**D**,**E**) After euthanizing the mice, the tumors of the mice were collected and weighed. (**F**) H&E staining of mouse tumor tissues and organs (heart, liver, spleen, lung, kidney). (**G**) Immunofluorescence staining of LC3B and P62 in tumors. Scale: 200 µM. * *p* < 0.05; ** *p* < 0.001; **** *p* < 0.00001; ns = not significant.

To evaluate the efficacy of IVM combined with MET in vivo for canine breast cancer, we used a xenograft breast cancer model in which canine breast tumor cells CMT-1211 were subcutaneously injected into the mammary fat pad of Balb/c mice and then randomly assigned for treatment (Figure 6A). During the whole treatment cycle, there was no significant difference in the body weight of mice in each group (Figure 6B). No obvious abnormality was found in the heart and kidney histopathological sections, indicating that drugs in each group had no apparent side effects on animals. The statistical results showed that the growth rate of xenograft tumors in mice treated with IVM and MET was slower than that in the control group (Figure 6C). Compared with the control group, the volume and weight of tumors treated with IVM and MET were significantly reduced, and H&E results showed that the IVM+MET treatment increased the cell necrosis in tumor tissue to a certain extent (Figure 6D–E). In addition to observing the effect of drugs on the tumor itself, we also paid attention to the inhibitory effect of drugs on the distant metastasis of tumor cells. Paraffin sections and H&E staining were performed on mouse tumors and essential organs. The results showed that the combined treatment group reduced lung and liver metastasis of breast cancer cells (Figure 6F). In addition, immunofluorescence detection was also performed on the tumor tissues of mice, and the results showed that the fluorescence intensity of LC3B combined with IVM and MET was significantly enhanced compared with the control group. In contrast, the fluorescence intensity of P62 was significantly decreased compared with the control group (Figure 6G). Taken together, these data suggest that IVM combined with MET has a favorable therapeutic effect on mouse breast cancer in vivo. In conclusion, we observed that IVM combined with MET had a significant inhibitory effect on tumor growth in canine breast tumor xenografts.

## 4. Discussion

Breast cancer develops primarily because of abnormal proliferation, inhibited apoptosis, and the accelerated invasion of breast cells. Inhibiting the proliferation and invasion of cancer cells and inducing their autophagy and death is one of the important mechanisms of drug therapy for breast tumors. Recent studies have shown that IVM can exert anti-tumor effects through multiple pathways, such as inhibiting the WNT-TCF signaling pathway, modulating PAK1, and inducing mitochondrial dysfunction [6,22,23]. The tumor-specific enhancement of IVM can also be attributed to the Warburg effect and high dependence on glycolysis in vivo hypoxic tumor environment [24]. As a first-line drug in treating diabetes, MET’s anti-cancer potential has also been confirmed by many studies, especially in breast cancer and other tumors with significant inhibitory effects. Recent studies have confirmed that MET can reduce the risk of tumor development and tumor-related mortality in patients with type 2 diabetes [25]. K. Saeki et al. found that metformin significantly inhibited tumor growth in xenografted metastatic CMGT cells [26]. Because of the anti-tumor effects of both drugs, this study, for the first time, used the two drugs in combination to evaluate the therapeutic effect of IVM combined with MET on canine breast cancer and explore its underlying mechanism.

In this study, the CCK-8 method was used to evaluate the growth of two breast cancer cell lines (CMT-1211, 4T1) treated with different drug groups. It was found that the cell viability of breast cancer cell lines treated with IVM or MET alone tended to decrease in a time- and dose-dependent manner. However, the activity of breast tumor cells in any single treatment group was higher than in the combined treatment group, indicating that the combination of IVM and MET had a strong synergistic effect. This can also be verified from scratch tests and invasion assay experiments.

In this study, transcriptome analysis was carried out based on the observation that drugs greatly decrease the activity of breast tumor cells. KEGG analysis showed that IVM combined with MET mainly involved the PI3K/AKT signaling pathway; GO analysis showed that combining the two drugs could affect the regulation of protein catabolism, polyamine metabolism, glycolysis, etc. These effects are directly or indirectly related to autophagy. The PI3K/AKT/mTOR pathway is closely associated with cell proliferation, differentiation, metabolism, autophagy, and other functions. Then, we explored the phosphorylation level of the PI3K/AKT/mTOR signaling pathway based on transcriptomic analysis. Western blot results showed that, compared with the single treatment group, the phosphorylation levels of the two cell pathway proteins showed a significantly decreased trend after combined treatment, indicating that IVM combined with MET could dramatically inhibit the activation of the PI3K/AKT/mTOR signaling pathway.

Autophagy plays a crucial role in tumor cells and the surrounding matrix, both of which influence tumor growth and drug resistance [27]. It is responsible for removing misfolded proteins and damaged organelles; otherwise, its dysfunction can result in abnormal cell function, ROS imbalance, inflammation, or defects in antigen presentation, making cells susceptible to malignant transformation [27]. Autophagy can inhibit tumor growth in certain cellular environments and induce cell death [28]. The accumulation of mitochondria in cells lacking key autophagy genes underscores the importance of mitochondria in tumor suppression, leading to ROS accumulation and DNA damage [29,30]. Similarly, damaged mitochondria are selectively targeted by autophagy, and their accumulation can result in oxidative stress and the loss of neuronal cells [31]. In this study, we observed through transmission electron microscopy that the combination of IVM and MET induces autophagy in breast tumor cells, leading to mitochondrial damage and dysfunction. This was evident as the mitochondria in the treatment group appeared swollen, with compromised double-membrane integrity, reduced or absent cristae, and a loose mitochondrial matrix. In contrast, the mitochondria of tumor cells in the control group maintained a relatively regular oval or rod shape, with clearly visible and neatly arranged cristae, indicating a normal structural and functional state. While autophagic adaptation can partially alleviate mitochondrial damage, excessive damage may ultimately lead to apoptosis [32].

Studies have shown that ROS, as a by-product of redox homeostasis, plays an important role in cell signaling processes and is essential for regulating the balance between autophagy and apoptosis in cancer cells following various drug treatments and gene modifications [33]. As signaling molecules, ROS participate in signal transduction pathways, regulating cell growth, differentiation, and survival, as well as contributing to inflammation and immune responses [34]. However, the excessive accumulation of ROS can damage DNA and cellular proteins, leading to a slowdown in cell metabolism, reduced activity, and even cell death [35]. Research has demonstrated that different forms of ROS serve various functions. For instance, ROS derived from NADPH oxidase can promote tumor cell proliferation, while ROS resulting from TIGAR deficiency have the opposite effect.

In our study, ROS levels in the IVM+MET group were significantly higher than in the other treatment groups. Furthermore, ROS levels in the IVM+MET group were notably reduced after the application of the ROS scavenger NAC. Reactive oxygen species detoxification is a crucial mechanism that cells use to maintain redox balance, eliminating excessive ROS through enzymatic and non-enzymatic systems to protect against oxidative damage. This process is vital for normal cellular functions and responses to stress.

Among various tumor types, the abnormal activation of the antioxidant transcription factor NRF2 provides strong evidence for the tumor-promoting effects of the cellular antioxidant program, with P62-mediated chelation leading to NRF2 accumulation [36]. Our results indicate that the expression level of P62 in the combined treatment group is lower than that in the control group, suggesting a weakened detoxification effect against reactive oxygen species. These findings imply that the interaction between autophagy and ROS may play a critical role in inhibiting the proliferation of breast tumor cells.

The PI3K/AKT/mTOR pathway is an important signaling pathway in cells, and it regulates cell growth, proliferation, metabolism, and survival. Cell growth and proliferation will be inhibited, which may lead to autophagic cell death.

Studies have shown that ROS can activate autophagy by inhibiting PI3K/AKT/mTOR [37,38]. LC3 is one of the signature proteins of autophagy formation [39], and the final mature autophagosome fuses with the lysosome under the action of the selective autophagy adapter protein p62 and degrades the autophagosome contents in the lysosome [40]. Our experiments showed that, in both the CMT-1211 and 4T1 cell lines, the IVM+MET group promoted the expression of LC3B and Beclin1 while decreasing the expression of P62 and Bcl-2. The application of NAC could partially mitigate this change.

Meanwhile, canine breast tumor cells CMT-1211 were subcutaneously injected into the breast fat pad of Balb/c mice using a xenograft breast cancer model. The results showed that the growth rate of xenograft tumors in mice treated with IVM combined with MET was slower than in the control group. IVM+MET treatment increased the necrosis of tumor cells to a certain extent. In addition to observing the effect of drugs on the tumor itself, we also paid attention to the inhibitory effect of drugs on the distant metastasis of tumor cells. We conducted paraffin sections and H&E staining on mouse tumors and essential organs, and the results showed that the combined drug group reduced the lung and liver metastasis of breast cancer cells. The immunofluorescence detection of mouse tumor tissues showed that the LC3B fluorescence intensity of IVM combined with MET was significantly enhanced compared with the control group. In contrast, P62 fluorescence intensity was significantly decreased compared with the control group. These data suggest that IVM combined with MET can significantly inhibit the growth of canine breast tumor xenografts in vivo.

## 5. Conclusions

In summary, the combination of IVM and MET promotes the production of reactive oxygen species, causes intracellular oxidative stress and mitochondrial dysfunction, and inhibits the activation of the PI3K/Akt/mTOR signaling pathway, thus inducing the necrosis and autophagy of breast cancer cells. These findings demonstrate a novel link between IVM combined with MET and autophagy mechanisms, suggesting that using IVM combined with MET as an autophagy inducer may constitute a novel treatment for breast cancer. In vivo, the experiments showed that ivermectin combined with metformin could significantly inhibit the growth of canine breast tumor xenografts. Future studies can further explore the specific details of this molecular mechanism and its potential clinical application value and provide new ideas and strategies for treating breast tumors.

## Data Availability

All the data used and analyzed for this study are available from the corresponding author upon reasonable request.
